# A Survey of Bioinformatics Database and Software Usage through Mining the Literature

**DOI:** 10.1371/journal.pone.0157989

**Published:** 2016-06-22

**Authors:** Geraint Duck, Goran Nenadic, Michele Filannino, Andy Brass, David L. Robertson, Robert Stevens

**Affiliations:** 1 School of Computer Science, The University of Manchester, Manchester, United Kingdom; 2 Manchester Institute of Biotechnology, The University of Manchester, Manchester, United Kingdom; 3 Computational and Evolutionary Biology, Faculty of Life Sciences, The University of Manchester, Manchester, United Kingdom; Macquarie University, AUSTRALIA

## Abstract

Computer-based resources are central to much, if not most, biological and medical research. However, while there is an ever expanding choice of bioinformatics resources to use, described within the biomedical literature, little work to date has provided an evaluation of the full range of availability or levels of usage of database and software resources. Here we use text mining to process the PubMed Central full-text corpus, identifying mentions of databases or software within the scientific literature. We provide an audit of the resources contained within the biomedical literature, and a comparison of their relative usage, both over time and between the sub-disciplines of bioinformatics, biology and medicine. We find that trends in resource usage differs between these domains. The bioinformatics literature emphasises novel resource development, while database and software usage within biology and medicine is more stable and conservative. Many resources are only mentioned in the bioinformatics literature, with a relatively small number making it out into general biology, and fewer still into the medical literature. In addition, many resources are seeing a steady decline in their usage (e.g., BLAST, SWISS-PROT), though some are instead seeing rapid growth (e.g., the GO, R). We find a striking imbalance in resource usage with the top 5% of resource names (133 names) accounting for 47% of total usage, and over 70% of resources extracted being only mentioned once each. While these results highlight the dynamic and creative nature of bioinformatics research they raise questions about software reuse, choice and the sharing of bioinformatics practice. Is it acceptable that so many resources are apparently never reused? Finally, our work is a step towards automated extraction of scientific method from text. We make the dataset generated by our study available under the CC0 license here: http://dx.doi.org/10.6084/m9.figshare.1281371.

## Introduction

Numerous database and software resources are published, used and mentioned within the medicine, biology and bioinformatics literature [1, 2]. Keeping up-to-date with bioinformatics resources is consequently difficult, but a necessary part of modern data management and analysis within biology and medicine. An up-to-date knowledge of available databases and software would be a valuable resource [3]. Previous attempts have been made to maintain accurate lists of available bioinformatics resources, though most have not been sufficiently comprehensive due to the slow process of manual curation, or specialised requirements for resource inclusion. For example, the Database of Databases (DoD) [4] and BioMed Central’s Databases catalog (http://databases.biomedcentral.com/) were previously established, but are no longer accessible. DBcat [5] was incorporated into a regular special issue from *Nucleic Acids Research* (NAR) [6], which lists databases which have been published at some point within that journal, and the Bioinformatics Links Directory [7], which is an associated special issue from the same journal, but focuses instead on web-services. In addition, some previous work has been relatively limited in scope, for example, either focused on only a specific domain (e.g., phylogenetics software [8]), or focused on specific journals (e.g., *Genome Biology* and *BMC Bioinformatics* [9]).

Automatic detection and extraction of bioinformatics resources from the literature would avoid the limitations of manual curation. Text mining has become established as a process with which to extract named entities—e.g., gene names [10], chemical names [11], species names [12], etc.—from the expansive literature now available. Past research has utilised text mining techniques for automatic software recognition, but generally only serves to help generate a repository of recognised resources, without performing any evaluation of their relative usage within the literature [13–15].

We use bioNerDS [9] to automatically extract database and software name mentions from the entire open-access set of PubMed Central full-text documents. As a result, we provide a survey of the usage of resources and trends in usage of these resources using a variety of metrics to enable us to start investigating the various stages of a resource’s life. This survey is undertaken in the context of biomedical literature, specifically, within *medicine*, *biology* and *bioinformatics*. In addition to an extensive evaluation of the available resources within bioinformatics, a full-scale survey of resource usage within bioinformatics and other associated domains is required to properly assess the current scale and state of resource usage, and help provide insight into scientific best-practice [8].

Best-practice provides a way to help identify the most appropriate method for a given task. However, the metrics to evaluate a given method can vary, including accuracy, recentness, public opinion, popularity, etc. [16]. The relative usage (or *popularity*) of a given resource can have an impact on best-practice, as if a resource is well used within a community, it must be considered *at least* sufficient for its intended purpose (i.e., *community-practice*). By automatically recognising and extracting database and software names in full-text literature articles, this will enable the repeatable discovery of trends in resource usage, enabling a comparison of which resources are used the most in which fields, and to see if other resources are poised to replace their competitors over-time.

## Materials and Methods

### bioNerDS Version 2

We make use of bioNerDS [9] to automatically extract database and software mentions from text. bioNerDS is a dictionary and rule-based resource recognition system, which uses a series of textual clues to recognise both old and new database and software names within the full-text literature.

BioNerDS recognises resource names by both matching terms against a pre-compiled dictionary of known names, and by combining several positive and negative rules within text. For example, once text has been preprocessed (tokenisation, sentence splitting, part-of-speech tagging, and dependency parsing), bioNerDS will identify version numbers, website URLs, and references. It will also identify both positive and negative keywords such as *database*, *software*, *tool* (all positive), *algorithm*, *method*, *approach* (all negative), within Hearst patterns [17] and by associating the “head” keywords with potential noun phrases using the dependency tree [18]. In addition, it applies a document wide score adjustment prior to thresholding which aids in recall by allowing numerous “weak” clues for a given potential resource to increase a resource’s overall score (rather than just relying on sentence level local clues). A “propagation” phase is then applied, which helps propagate document level matches to the mention level. Finally, scores from individual rules are adjusted based on character case and length (e.g., for acronyms), and a minimum threshold is applied before the final results are produced. Various aspects of the bioNerDS system both help reduce false positive mentions, while others help improve final recall. For complete details of the original bioNerDS system, please refer to [9].

Before applying bioNerDS to PubMed Central, we have made several changes to bioNerDS to further improve the resulting recognition accuracy and produce bioNerDS version two:

1. A new resource list based on the ontology listings at the NCBO BioPortal [19], which contains 358 ontology names, has been added. We have also updated all the database and software name lists used within bioNerDS’ dictionary to the 16th of December, 2013. These changes have resulted in 1,997 additional dictionary entries over the previous release, resulting in 8,214 total entries, and 7,727 total unique name variants (Table 1).
2. The previous acronym detection to filter out known incorrect matches has been extended. Specifically, if an acronym is detected in text, and its expanded form is in the dictionary, then the mention is only accepted if its detected expanded form (identified in text using BADREX [20]) matches the expected expansion. This comparison is performed using fuzzy regular expressions in FREJ (http://frej.sourceforge.net/).
3. Additional restrictions on some head-word based matches have been applied—this helps reduce the false positives generated from ambiguity in these head terms (specifically *program*, *system*, *project* and *service*).

**Table 1:** bioNerDS v2 dictionary breakdown.

Type	Entries	URL	Still Accessible?
DB	194	databases.biomedcentral.com	N
SW	262	www.bioinformatik.de	N
PK	1013	www.bioconductor.org	Y
SW	2101	www.bioinformatics.ca/links_directory/	Y
SW	385	evolution.genetics.washington.edu/phylip/software.html	Y
DB	506	www.ebi.ac.uk/miriam/main/	Y
DB	1510	www.oxfordjournals.org/nar/database/a/	Y
SW	134	www.netsci.org/Resources/Software/Bioinform/index.html	Y
DB	358	http://bioportal.bioontology.org/ontologies	Y
SW	38	www.bioinf.manchester.ac.uk/recombination/programs.shtml	Y
SW	1196	en.wikipedia.org/wiki/Wiki/<various>	Y
—	517	Manually generated entries	-

Database and software URLs from which the bioNerDS database and software name dictionary is comprised, with 8,214 total entries. DB = databases; SW = software; PK = packages; data correct as of 16th December, 2013.

### bioNerDS version 2 post-processing filter

In addition to bioNerDS itself, a machine-learning based filter has been built and applied over the updated bioNerDS software, with the aim of automatically discarding false positive mentions. This was implemented as bioNerDS frequently generated false positive results within the “long-tail” of resource mentions. A similar approach has been used previously, where a machine learning post-processing filter has been applied to help improve system precision of resource mention detection [14].

As each mention extracted by bioNerDS usually matches more than one rule, we use the information about which rules were matched in order to filter out likely false positives, and consequently improve precision. As such, we used 17 binary features (one feature per rule), where each is enabled if the corresponding rule was matched for the particular mention. We also added a further feature that represents the total number of rules matched per expression. We intentionally avoid the use of lexical and morpho-syntactic features, thus forcing the classifier to learn exclusively on the basis of the system’s associated rules, mitigating over-fitting.

**Fig 1:**
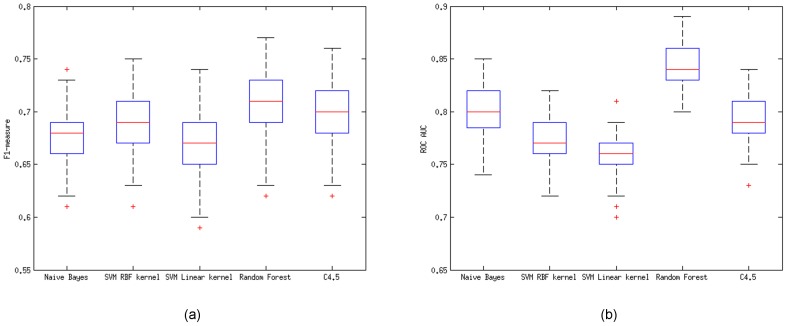
Model selection results across five different machine-learning classifiers, using 10x10-fold cross-validation. The box indicates the upper/lower quartiles, the horizontal line inside each of them shows the median value, while the dotted crossbars indicate the maximum/minimum values. Both the *F*_*β* = 1_ measure (a) and ROC Area Under the Curve (b) comparisons indicate that Random Forest provides the best performance.

We compared five different machine learning classifiers: Naïve Bayes, SVM with RBF kernel, SVM with linear kernel, Random Forest and Decision Tree (C4.5). We used both *F*_*β* = 1_ measure (Fig 1a) and Area Under the ROC Curve (AUC; Fig 1b) [21]. Analysis was performed on the 60 full-text articles initially used for bioNerDS development and evaluation [9], which have been shuffled 10 times with a 10-fold cross-validation technique applied (Table 2). The analysis is statistically significant (*p* = 2.8661 * 10^−115^ with ANOVA test), and indicates Random Forest to be the best performing model (Precision: 0.80, Recall: 0.64, F-score: 0.71). This classifier has been subsequently trained on all the available annotated data and integrated into bioNerDS as a post-processing filter.

**Table 2:** Model selection results.

	Precision		Recall		*F*_*β* = 1_		AUC	
Classifiers	avg	(std)	avg	(std)	avg	(std)	avg	(std)
Naïve Bayes	.67	(.028)	**.68**	(.032)	.68	(.026)	.80	(.022)
SVM Linear	.77	(.033)	.63	(.034)	.69	(.027)	.77	(.018)
SVM RBF	.72	(.044)	.63	(.036)	.67	(.028)	.76	(.019)
Random Forest	**.80**	(.035)	.64	(.037)	**.71**	(.030)	**.84**	(.018)
C4.5	.79	(.037)	.62	(.042)	.70	(.031)	.80	(.020)

Model selection results averaged over 10 runs (10 cross-fold validation per run). Random Forest model proved to be better in terms of precision and AUC measure, whereas Naïve Bayes is better with respect to recall. In terms of *F*_*β* = 1_ measure Random Forest and C4.5 are almost equal.

### Quality of extracted resource names by bioNerDS v2

A new document set was annotated for evaluation of bioNerDS version 2 [22]. This new set contains 25 full-text articles, randomly selected from *BMC Bioinformatics* and *PLoS Computational Biology*, which have been annotated according to the guidelines previously published [23]. The random selection was performed by assigning each potential article an integer from *1 to n*, and then by using random.org’s *sequence generator* with the same range (https://www.random.org/sequences/). This new set contained 1,479 database and software mentions, with 301 unique resource names. Although this set is potentially biased towards bioinformatics’ articles, previous experiments have shown either comparable or more favourable results when testing on alternative corpora (e.g., on *Genome Biology* articles) [9]—most likely because other domains (e.g., biology, medicine) have fewer total resources making recognition more straightforward.

Table 3 shows the results of the different bioNerDS versions evaluated with respect to this new evaluation set. Note that strict scores require offsets to match exactly between both the gold and system annotations, whereas lenient matches are a true positive as long as there is at least *some* offset overlap between annotations. A strict match is always also a lenient match.

**Table 3:** bioNerDS version 2: Evaluation.

	Precision	Recall	*F*_*β* = 1_
bioNerDS (version 1)	.61 (.73)	.59 (.71)	.60 (.72)
bioNerDS improved (version 2)	.66 (.69)	**.69** (.72)	**.67** (.70)
bioNerDS improved with post-processing filter	**.79** (.82)	.54 (.56)	.64 (.67)

Evaluation scores for the previous version of bioNerDS, the updated version presented within this paper, and the updated version including the new post-processing filter. Figures in brackets refer to lenient scores.

The improved version provides a far smaller (halved in most cases) gap between lenient and strict scores, and outperforms its original version with respect to precision, recall and *F*_*β* = 1_ measure. The use of the machine learning-based filter had a strong positive impact on precision, but recall is negatively affected, leading to a small drop in terms of *F*_*β* = 1_ measure. These results additionally outperform a previously published machine learning approach for resource recognition, which had reported a strict f-score of 63% (and an associated lenient f-score of 70%) [22].

### Resource name extraction

We have applied bioNerDS to 713,634 full-text articles taken from the PubMed Central open-access corpus, as downloaded in December 2013. Here we used the complete and unfiltered set of PubMed Central articles, as provided on their ftp server (ftp://ftp.ncbi.nlm.nih.gov/pub/pmc/). We note that 135 of these articles could not be correctly processed, due to resource constraints primarily resulting from the computational complexity of dependency parsing. We ensured a minimum confidence in the extracted mentions by removing all mentions for which bioNerDS’ post-processing filter was at least 80% confident were false positive hits, providing an appropriate trade-off between precision and recall.

We characterise various sub-domains (medicine, biology and bioinformatics) by splitting the corpus into these three sub-corpora. This is done on a per-journal basis, dependent on the MeSH terms assigned to a given journal name through “Broad Subject Terms for Indexed Journals” searchable via the NLM Catalog (https://www.ncbi.nlm.nih.gov/nlmcatalog). These terms aim to describe a journal’s overall scope, but are only assigned to MEDLINE journals. It is important to note that this hierarchy makes the bioinformatics domain a strict subset of the biology domain. Corpora summary statistics are provided in Table 4.

**Table 4:** Corpora Summaries.

	Full PMC	Medicine	Biology	Bioinformatics
Associated MeSH Term	-	H02.403	H01.158.273	H01.158.273.180
Total Journal Names	3,849	650	430	67
Total Documents	713,634	152,464	126,376	25,851

Overview of the different corpora used throughout this paper, as separated through associated journal MeSH terms.

Although this provides an appropriate way to compare these domains (as any sub-domain of a MeSH term will be included in each case), it can still exclude journals that would be appropriate for inclusion (and which indeed have associated MeSH terms), but currently are not, as they are located along a different “branch” of the hierarchy. For example, only the full PMC corpus included mentions from *Nucleic Acids Research* as it has “Nucleic Acids” as an associated MeSH term (under “Chemicals and Drugs Category”), which is not a sub-term of biology, medicine or bioinformatics (under “Disciplines and Occupations Category”).

### Journal and resource clustering analysis

We make use of singular value decomposition (SVD) to automatically cluster our bioNerDS generated data. This enables us to investigate any separation between journals based on the resources mentioned within, and conversely, investigate a separation between resources based on the journals in which they are mentioned. This analysis is based on an underlying matrix of resources in one direction against journals in the other, with each cell containing the number of document level mentions for that resource in a given journal.

We are unable to use principal component analysis (PCA), due to the large sparse data matrix we generate—this would otherwise require us to normalise the mean counts to zero. With SVD we are still able to extract the eigenvectors and eigenvalues from our sparse matrix. We can then use the most significant vectors as a separation of our data. We note that because we are using SVD rather than PCA, the most significant eigenvector is a separation of “scale”, and so we exclude this from our analysis (in each case the implied ordering by the top vector reasonably equates to the total mention count orders discussed elsewhere in the paper, and accounts for 80% of the variation seen within the data).

### Section analysis

In order to perform zoning to automatically identify the various document sections, we extend our previous method section recognition approach [16] to also differentiate between the introduction, results/discussion and conclusion. We combine the results and discussion sections into a single category as they are often grouped together within journal articles. This approach uses a series of automatically extracted and manually extended regular expressions to identify the various section headings within articles. This achieved sufficient accuracy (recall of 76.4% and precision of 88.9%) within our manually evaluated test corpus of 100 randomly selected articles.

This method was then applied to our entire set of full-text PMC articles to determine the start and end offsets of each document section, from which we could filter the mention offsets as returned by bioNerDS.

### Caption analysis

To evaluate resource usage within the captions of articles, we first processed the XML for each article within our full PMC corpus. From this, we extracted all the text that was located within <caption> and </caption> tags and directly passed this to bioNerDS for resource recognition, again using a confidence threshold of 80% during post-processing. From our 713,634 total articles, 550,400 contained at least one caption tag and 78 of these could not be successfully processed.

## Results

bioNerDS extracted a total of 5,411,968 resource mentions from the full PubMed Central biomedical literature corpus (713,634 full-text articles). 61.2% of the documents contained at least one resource mention, with 1,356,951 total document level mentions. This reduces to 3,926,176 total mentions (1,279,111 document mentions) once we apply the minimum post-processing filtering confidence threshold of 80% (we continue to use this threshold throughout the remainder of our results analysis).

We use these results from bioNerDS to perform the following analyses:

1. Compare resource usage between this full corpus, and between medicine, biology and bioinformatics sub-corpora.
2. Contrast how the relative usage of different resources has changed over each of the last 14 years.
3. Compare the usage levels of databases and software between various common journals, and highlight “outlier” journals.
4. Evaluate claims that only very few resources make up the majority of mentions, while most resources are rarely (if ever) used [2].
5. Analyse variations in resource mentions between the four primary document sections (i.e., introduction, methods, results/discussion and conclusion).
6. Analyse document captions (figures, tables, supplementary data, etc.) to see how resource usage in these differs from the main-text.

### Resource mentions in medicine, biology and bioinformatics

As would be expected, our *bioinformatics* sub-corpus contained a relatively large number of mentions given its smaller corpus size (797,501 total and 172,099 document level mentions from 25,851 documents). By comparison, the *biology* corpus (1,628,790 total and 400,691 document level from 126,376 documents), which has a higher number of mentions; and to the *medicine* corpus (663,988 total and 170,145 document mentions from 152,464 documents), which has fewer mentions despite being a larger corpus. *Bioinformatics* has the highest proportion of mentions with a mean of 30.8 mentions per document, followed by *biology* and then *medicine*, with means of 12.9 and 4.4 mentions per document, respectively. The mean for the entire PMC corpus was 5.5 mentions per document.

We first compared the numbers of unique resource names extracted within each corpus, calculating the overlaps (intersections) between each. 65% of the unique names within *medicine* were unique to that corpus, the remainder was evenly split between *biology*, and *biology* and *bioinformatics* combined. There were none just within *bioinformatics* as that corpus is a strict subset of *biology*. 38% of names within the *biology* corpus were not mentioned in either other corpus, with 41% being additionally mentioned within *bioinformatics* and 11% within *medicine*; 11% were mentioned within both *medicine* and *bioinformatics*. Finally, names within the *bioinformatics* corpus were split 79% within just *biology*, and the other 21% within both *biology* and *medicine*.

### Top resource mentions

Tables 5 to 8 provide the top ten most mentioned resources at both the document and mention level within each of our corpora. In both *biology* and *bioinformatics*, many well established resources appear, with a stronger focus on data access (databases) rather than data analysis (software). This is perhaps just because there are fewer database than software names within the domain. Established data resources such as GenBank, UniProt, GO, KEGG and PDB all feature, though so too do the generic analysis tools BLAST and R. Within *bioinformatics*, GO has more mentions than R, whereas R has more document level mentions than GO. This could highlight that GO is used frequently for annotation within a document resulting in a high mentions per document count.

**Table 5:** Top ten terms in Bioinformatics.

Mention Level		Document Level	
GO	2.08	R	0.29
R	1.17	GO	0.19
BLAST	0.62	BLAST	0.16
PDB	0.43	GenBank	0.13
KEGG	0.43	GEO	0.09
GenBank	0.35	KEGG	0.09
Ensembl	0.24	PDB	0.08
GEO	0.24	Ensembl	0.06
Pfam	0.20	Cluster	0.06
Cluster	0.18	UniProt	0.05

The mention level numbers provide the average mentions per document, and the document level number provides the fraction of the *bioinformatics* corpus to contain at least a single mention of that resource.

**Table 6:** Top ten terms in Biology.

Mention Level		Document Level	
R	0.66	R	0.18
GO	0.57	GenBank	0.09
GenBank	0.24	BLAST	0.07
BLAST	0.21	GO	0.06
PDB	0.15	PDB	0.04
KEGG	0.11	GEO	0.03
GEO	0.08	SPSS	0.03
Ensembl	0.08	ClustalW	0.03
ABA	0.07	KEGG	0.03
Cluster	0.06	Cluster	0.02

The mention level numbers provide the average mentions per document, and the document level number provides the fraction of the *biology* corpus to contain at least a single mention of that resource.

**Table 7:** Top ten terms in Medicine.

Mention Level		Document Level	
SPSS	0.18	SPSS	0.12
R	0.16	R	0.06
Interleukin	0.04	Stata	0.02
ECG	0.04	PubMed	0.02
PubMed	0.03	Effective	0.01
GO	0.03	GenBank	0.01
Stata	0.03	ECG	0.01
ECG	0.03	MEDLINE	0.01
GenBank	0.03	ICD	0.01
Healthcare	0.02	GraphPad Prism	0.01

The mention level numbers provide the average mentions per document, and the document level number provides the fraction of the *medicine* corpus to contain at least a single mention of that resource.

**Table 8:** Top ten terms in the Full PMC Corpus.

Mention Level		Document Level	
R	0.43	R	0.14
GO	0.16	SPSS	0.09
SPSS	0.13	GenBank	0.04
GenBank	0.10	BLAST	0.03
PDB	0.07	GO	0.02
BLAST	0.07	PDB	0.02
KEGG	0.04	GraphPad Prism	0.02
PubMed	0.04	Stata	0.02
GEO	0.03	PubMed	0.02
ECG	0.03	SMART	0.02

The mention level numbers provide the average mentions per document, and the document level number provides the fraction of the *Full PMC* corpus to contain at least a single mention of that resource.

Our *medicine* corpus shows a slightly different story—there are more mentions that may stem from false-positive mentions (e.g., healthcare, ECG, effective, etc.); this is likely to be an artefact of bioNerDS being trained on bioinformatics rather than medical text. That said, medicine shows a clear preference for SPSS and Stata over R for statistical processing (with double the mean number of SPSS document level mentions compared to R; Table 7), and also shows a strong affinity for retrieval of the literature, with PubMed and MEDLINE in its top ten resource list. Although these two terms can be often used interchangeably within the literature, as they are different resources (e.g., PubMed provides access to MEDLINE *in addition to* other resources; https://www.nlm.nih.gov/pubs/factsheets/dif_med_pub.html), we keep them separate within our analyses. Finally, Table 8 provides the result for the full PMC corpus, which is a combination of the preceding tables already discussed (especially given the high resources to documents ratio within our *bioinformatics* corpus).

### Temporal analysis of top resources

**Fig 2:**
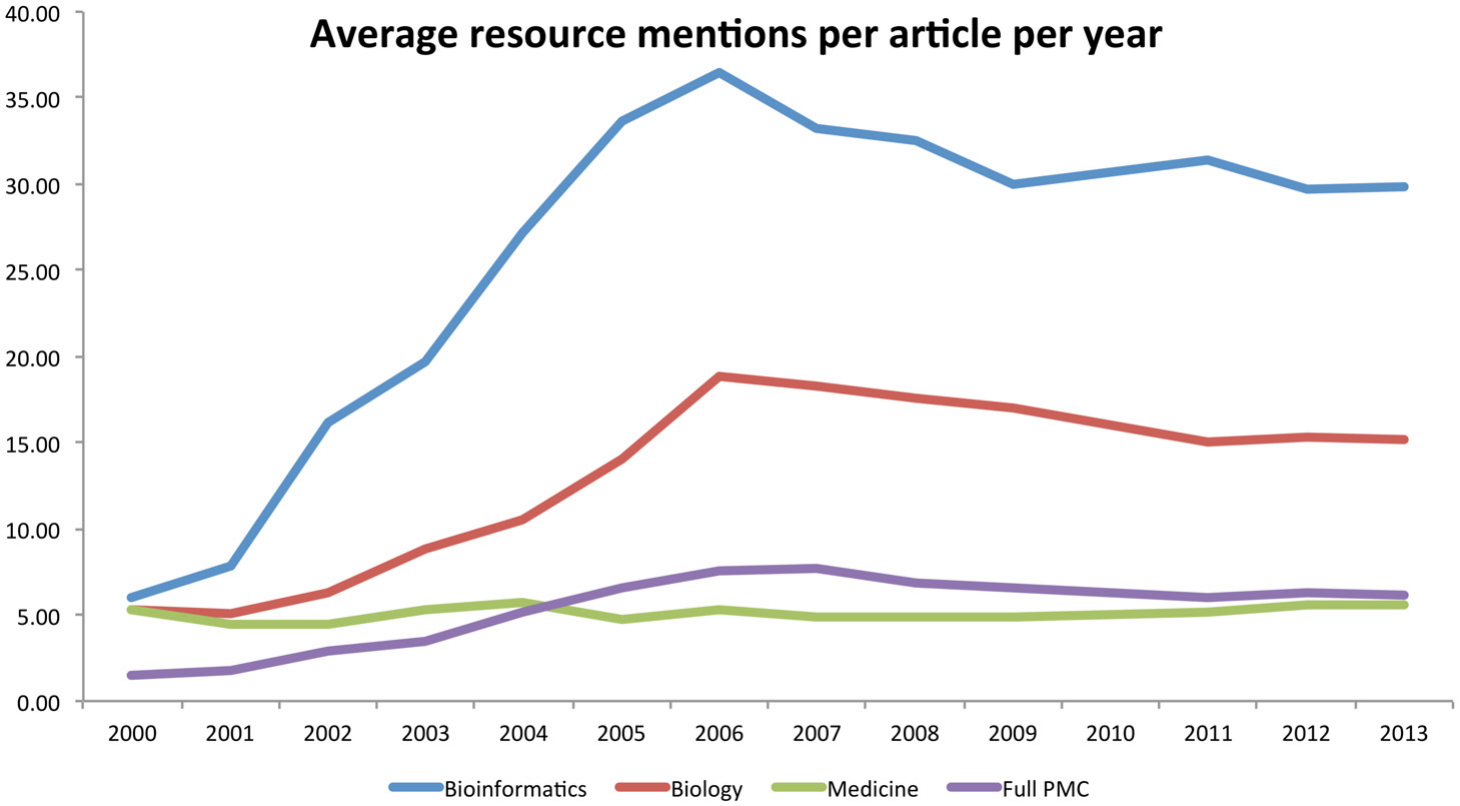
Average number of resource mentions per article in each document corpus evaluated over time.

**Fig 3:**
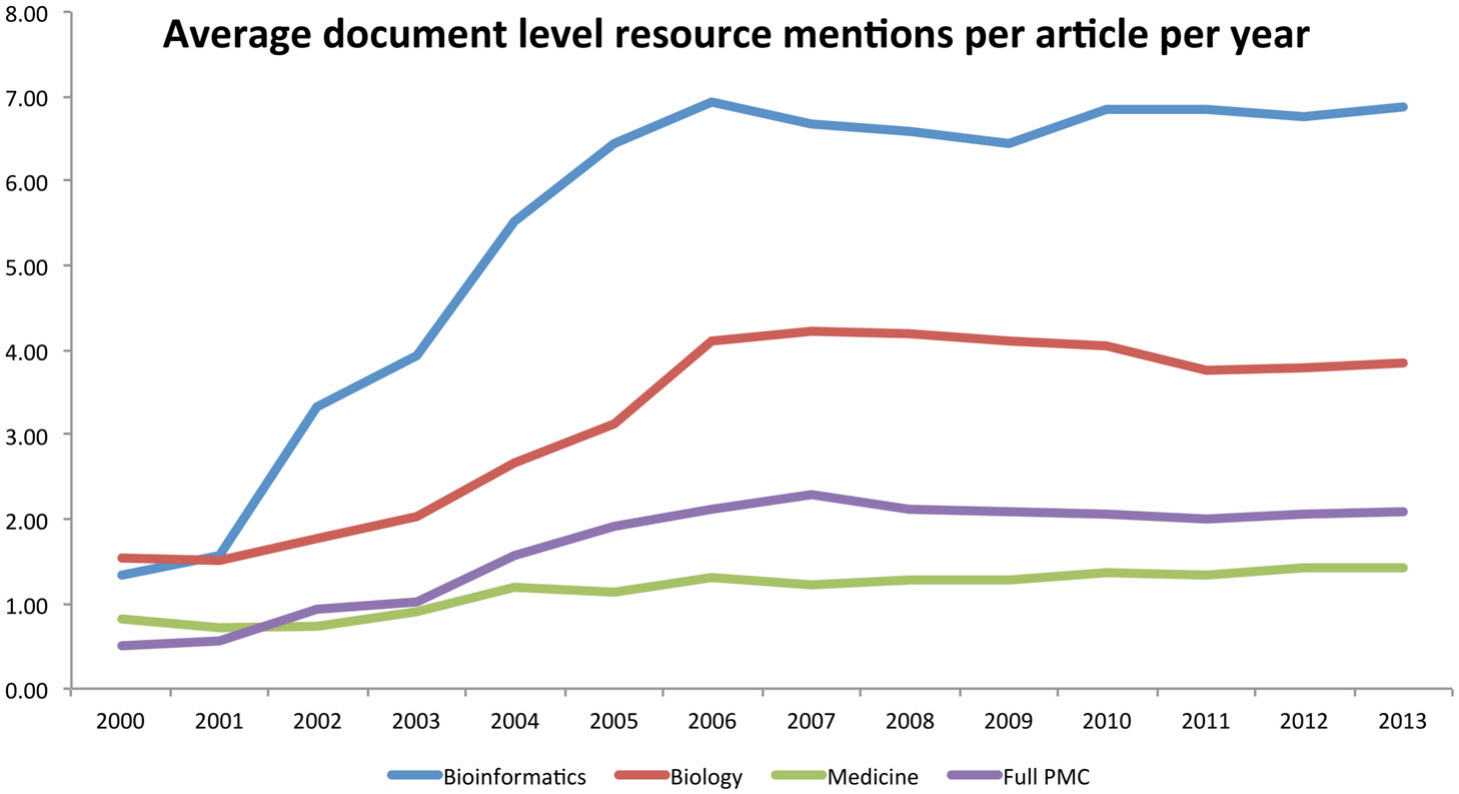
Average number of document level resource mentions per article in each document corpus evaluated over time.

**Fig 4:**
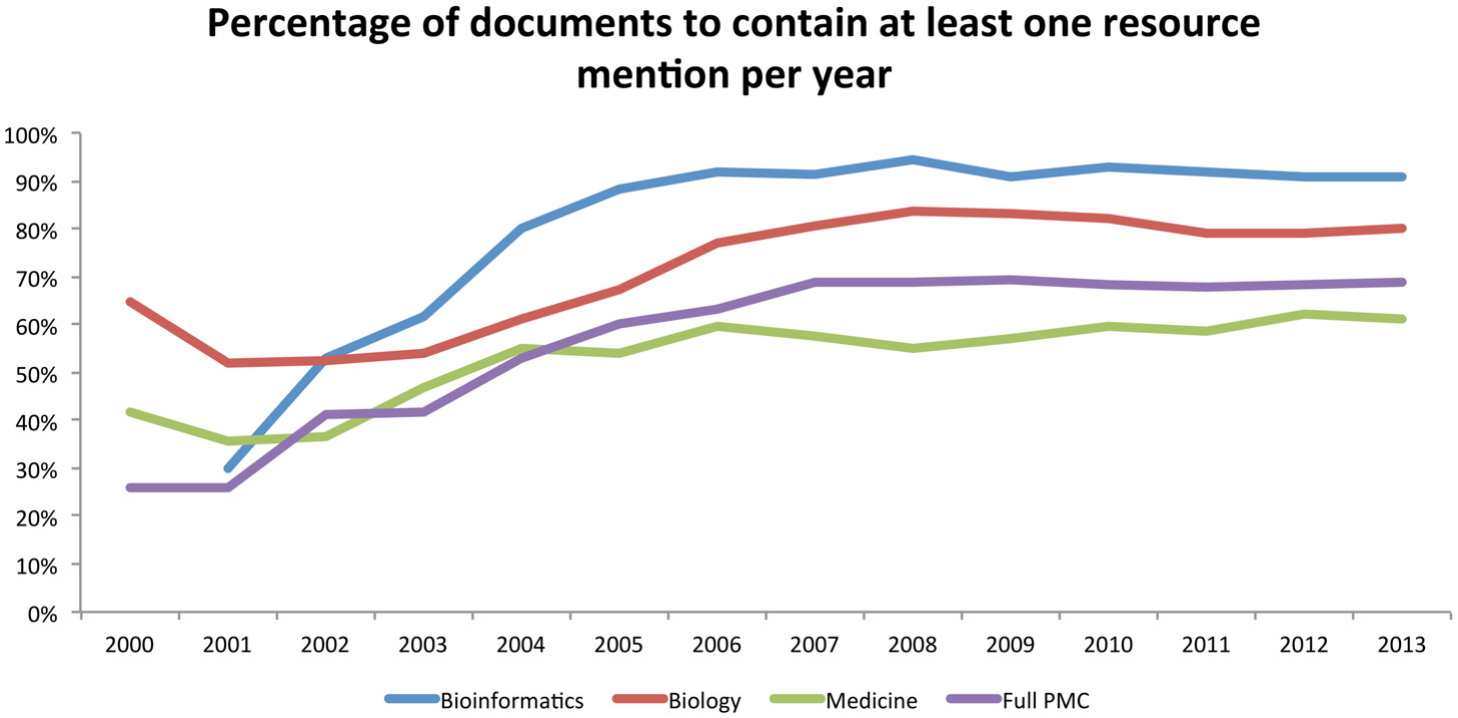
The percentage of articles within each document corpus to contain at least one extracted resource mention, as evaluated over time.

To evaluate the changes in resource usage within each field over time, we grouped the extracted mentions into years based on the publication year of the article from which each mention was extracted. We ignore mentions prior to the year 2000, as there are insufficient data in some of our domains (especially *bioinformatics*). Specifically, we provide graphs detailing the mean number of mentions and mean number of document mentions per year in each of our corpora (Figs 2 and 3; as normalised by the total article counts in each year); and the percentage of the total articles within each corpus that contained at least one resource mention (Fig 4). In general, these graphs show that there was a rapid increase in database and software usage between the years 2000 and 2006, after which usage has plateaued—this appears true for all of our corpora. The clearest diversion from this pattern is within the mean total mentions graph, which shows a slight decline for *bioinformatics* and *biology* since 2006. Aside from this, as would be expected, *bioinformatics* shows consistently higher numbers of resource mentions, followed by *biology* and then *medicine*. This agrees with the total numbers reported earlier in the paper. Medicine shows numbers lower than the mean for the full PMC corpus, which can be explained by the overwhelming numbers within *biology* and *bioinformatics*, as well as *medicine* being likely to contain other article types not requiring direct computational analysis (e.g., Case Reports, Population Studies, etc.).

**Fig 5:**
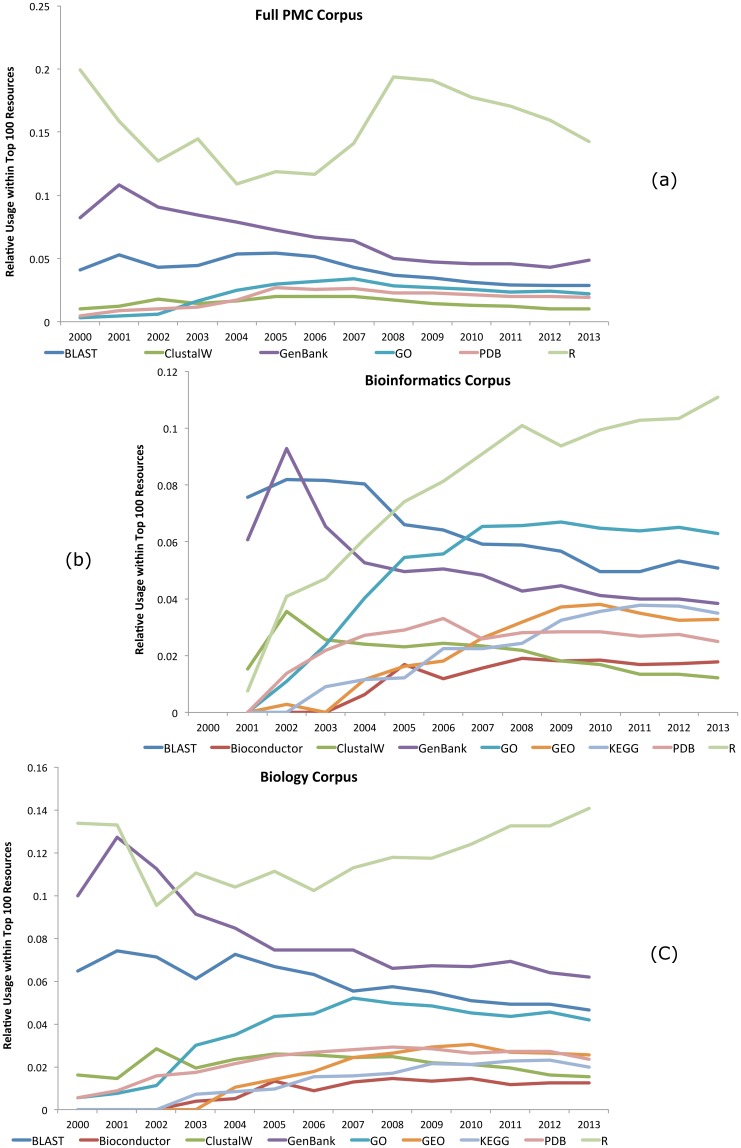
The relative usage of several key resources within the top 100 mentioned resources (document level), for each of our corpora, as calculated over time. No graph is provided for our *medicine* corpus as the relative usage numbers within that corpus were low.

In order to analyse how various database and software resources have been used over time, we selected several well-known and established resources, and extracted the numbers of document level mentions of each of these resources in the years 2000 to 2013 from within the top 100 resources in each year, for each of our corpora. Once each mention count is divided by the total mentions of the top 100 resources in each case, this provides us with an indication of the relative usage of the resource within each field, and in particular, how stable that usage is within the top 100 resources. Fig 5 provides the results of this analysis for each of our corpora. We provide no graph for our *medicine* corpus, however, as the relative usage numbers within that corpus were understandably low for recognised bioinformatics resources. Of interest, however, is that it has seen a steady increase in the relative number of mentions of SPSS, as well as slight increases in mentions of PubMed and ClinicalTrials.gov, while R and MEDLINE have remained stable.

Within our full PMC corpus, R has seen high levels of fluctuation in relative usage, with a peak in 2008. This is in contrast to both our *bioinformatics* and *biology* corpora where it has seen continued growth, though the growth is more substantial within *bioinformatics*. In all four of our corpora, GenBank’s relative usage has been in decline since 2001 and the same is true for both BLAST and ClustalW, although less substantial. This could be a result of the increase in more specialised/alternative alignment programs as well as the continued specialisation of the bioinformatics domain, or because papers mention (or cite) resources less frequently as they become more ubiquitous (i.e., assumed knowledge).

The remaining resources within Fig 5a show little variation (and we do not plot them all for this reason), though these can be examined in more detail within our other two more specific corpora. The GO shows a strong increase in relative use between 2000 and 2007 in both *bioinformatics* and *biology*, though it has since settled. Bioconductor additionally shows an increase prior to about 2008, and there is a similar story for PDB. KEGG shows a stronger increase in *bioinformatics* than *biology*, and the relative usage of GEO increases within both datasets. In addition, UniProt’s relative usage has been steadily increasing, while SWISS-PROT usage has been steadily decreasing in both the *bioinformatics* and *biology* corpora, which makes sense as UniProt is designed to incorporate and replace SWISS-PROT. Finally, mentions of Microsoft Excel have remained stable in our *bioinformatics* and *medicine* corpora, but suffered a unexpected decrease in 2006 within both our *biology* set and our full PMC set (note that the decrease in the full PMC set could be as a direct result of the decrease in the *biology* set).

**Fig 6:**
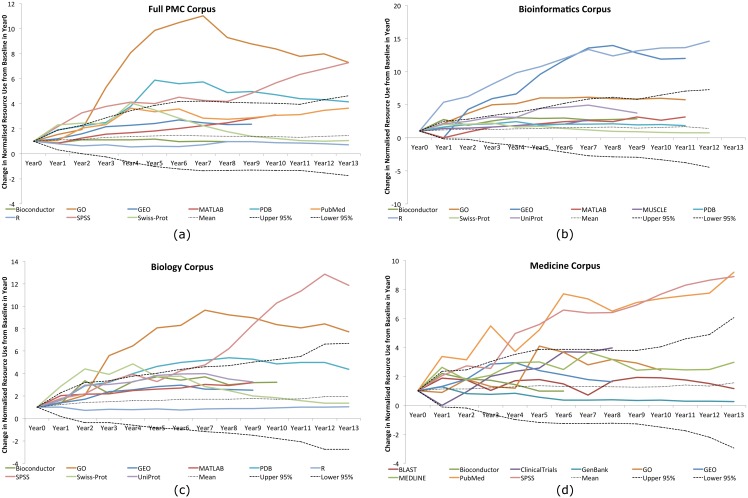
The upper and lower 95% confidence bounds in normalised relative change for several key resources in each of our corpora.

To evaluate the significance of the change in relative resource usages described above, we normalised each resource to its baseline by dividing each year value by that resource’s relative usage in *Year0* (i.e., the first year in which we see it within the top 100 resources for a given corpus), and compare the change of a given year from Year0 to that of a Gaussian distribution, as modelled on our underlying data using a random walk process in the same way that we have done previously [9]. Divergence from the 95% standard deviation confidence bounds would suggest significant changes in usage. Fig 6 provides the results for each of our corpora, highlighting some of the more interesting resources in each case.

Within the full PMC corpus, there has been significant increase in usage of the GO, the PDB and SPSS, with sudden growth and then a decline in SWISS-PROT. PubMed additionally showed a significant usage increase, but has since levelled out. *Bioinformatics* has seen rapid uptake in R and GEO, and a high uptake in the GO and MUSCLE. In contrast, *biology* has favoured SPSS and the GO with insignificant changes in R and little in GEO. It has also seen significant initial growth in SWISS-PROT and the PDB. Finally, *medicine* has seen a significant usage increase in both PubMed and SPSS, with modest increases in the GO, MEDLINE and ClinicalTrials.gov.

To explore the “rate” of change in resource usage within each corpus, for each resource we calculated the sum of the relative frequencies within the top 100 resource mentions within each year (years 2000 to 2013 inclusive), and plotted this against the sum of the absolute differences for each resource between each year (Δ*Σ*). Specifically, for each resource (*x*) in a given year (*y*) [9]:

$$\Sigma \Delta =\sum_{y=2000}^{2013}\left|x^{y+1}-x^{y}\right|$$ (1)

As such, a high relative frequency would imply that the resource has seen consistently high usage over time, whereas a high absolute difference sum (Δ*Σ*) would imply that the resource has seen a high fluctuation in its usage levels.

**Fig 7:**
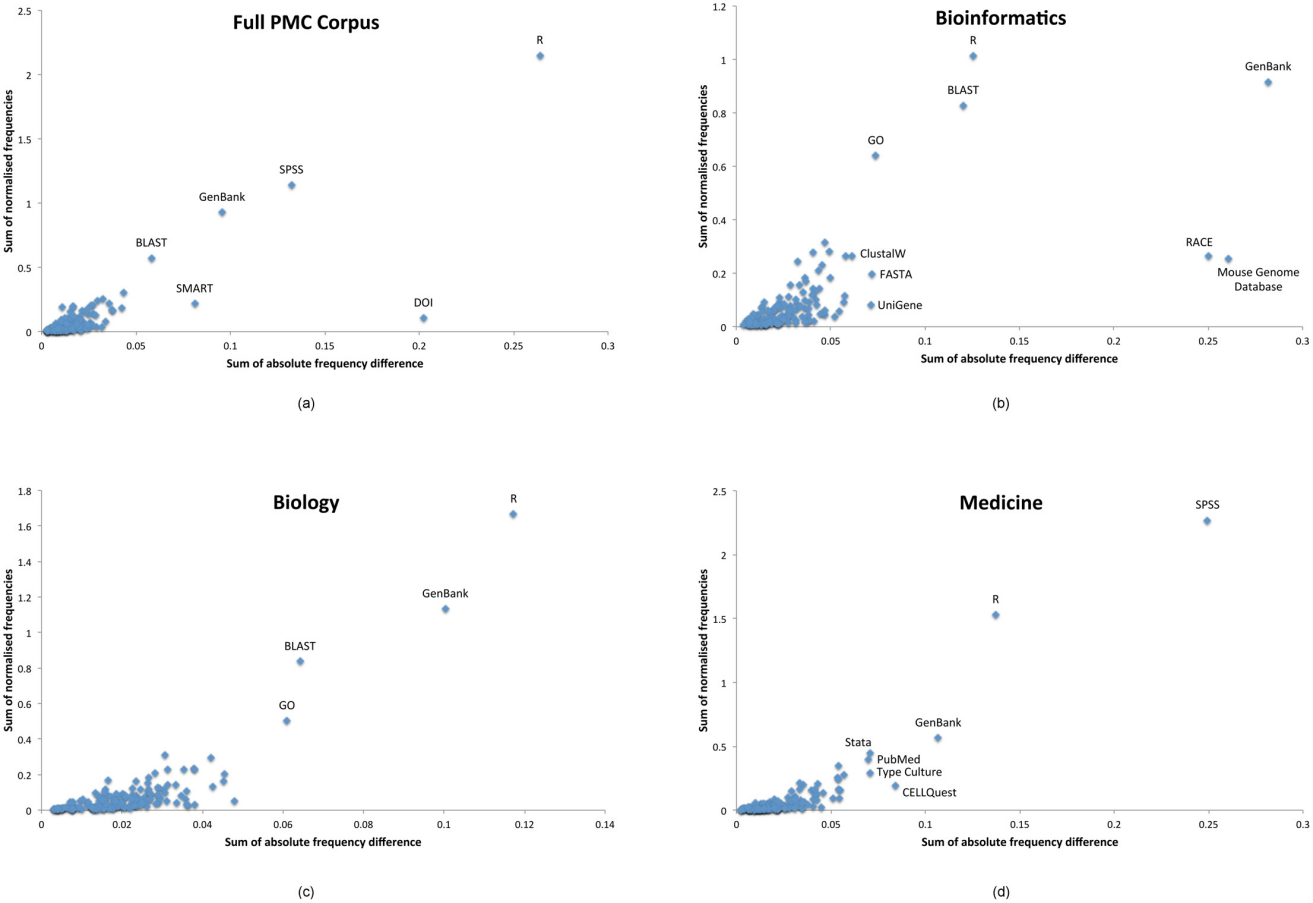
Relative usage variation within the top 100 resources for each of our corpora. We plot the sum of the normalised frequencies (y-axis; relative resource usage), against the sum of the absolute differences (x-axis; usage variation), with interesting outliers labelled. Data based on resource mentions extracted in the period 2000–2013 inclusive. We filtered out mentions only seen in a single year.

Within all four of our corpora (Fig 7), R shows high levels of both usage and variation (though less variation within *bioinformatics*), which substantiates the general increase in its usage we have already seen. GenBank also features highly in all four of our corpora, though it is more pronounced in *bioinformatics* and *biology*. BLAST is shown in all but our *medicine* dataset, which instead has SPSS as both the resource with the highest usage and highest variation. The Gene Ontology (GO) has a relatively high level of usage within *bioinformatics* and high variation within *biology*. Finally, *bioinformatics* has high variation in usage of both RACE and the Mouse Genome Database. In each of these cases, it is because they saw high initial usage within the first few years of our dataset, but none (within the top 100 resources) thereafter—so the high rate of change is a rapid relative decrease in usage.

### Temporal analysis of “medium-usage” resources

We wanted to look into resources that are well established within a bioinformatics’ sub-domain, but which are not pervasive enough to be used within the wider domain. We did this by filtering down our total resource mentions to only those which have at least one document mention *each year* from 2000 to present with no gaps. We note that this is a particularly strict criterion as, for example, a resource might be initially published in one year, and then require a year or two before it starts getting used/established; but this helps provide a suitable definition of an established resource—that it is consistently used once we first identify it within the literature. In order to remove the top resources previously discussed, we further removed resources with mentions in 2000 (i.e., those that have ‘always existed’). We note that this removes 138 resource names from our analysis, and results in 44 out of the top 50 resources (based on document level counts) being filtered out, leaving: Bioconductor, ClinicalTrials.gov, GEO, ImageJ, RefSeq and UniProt, all of which have become established resources since the year 2000. Finally, we create two datasets: one without resources that still exist in 2013 (those which have not been reported), and one which includes those mentioned in 2013. It is possible that this filtering step removes some particularly pervasive “medium” resources, however we would argue that any resource mentioned consistently since 2000 is a top, rather than medium, resource.

**Fig 8:**
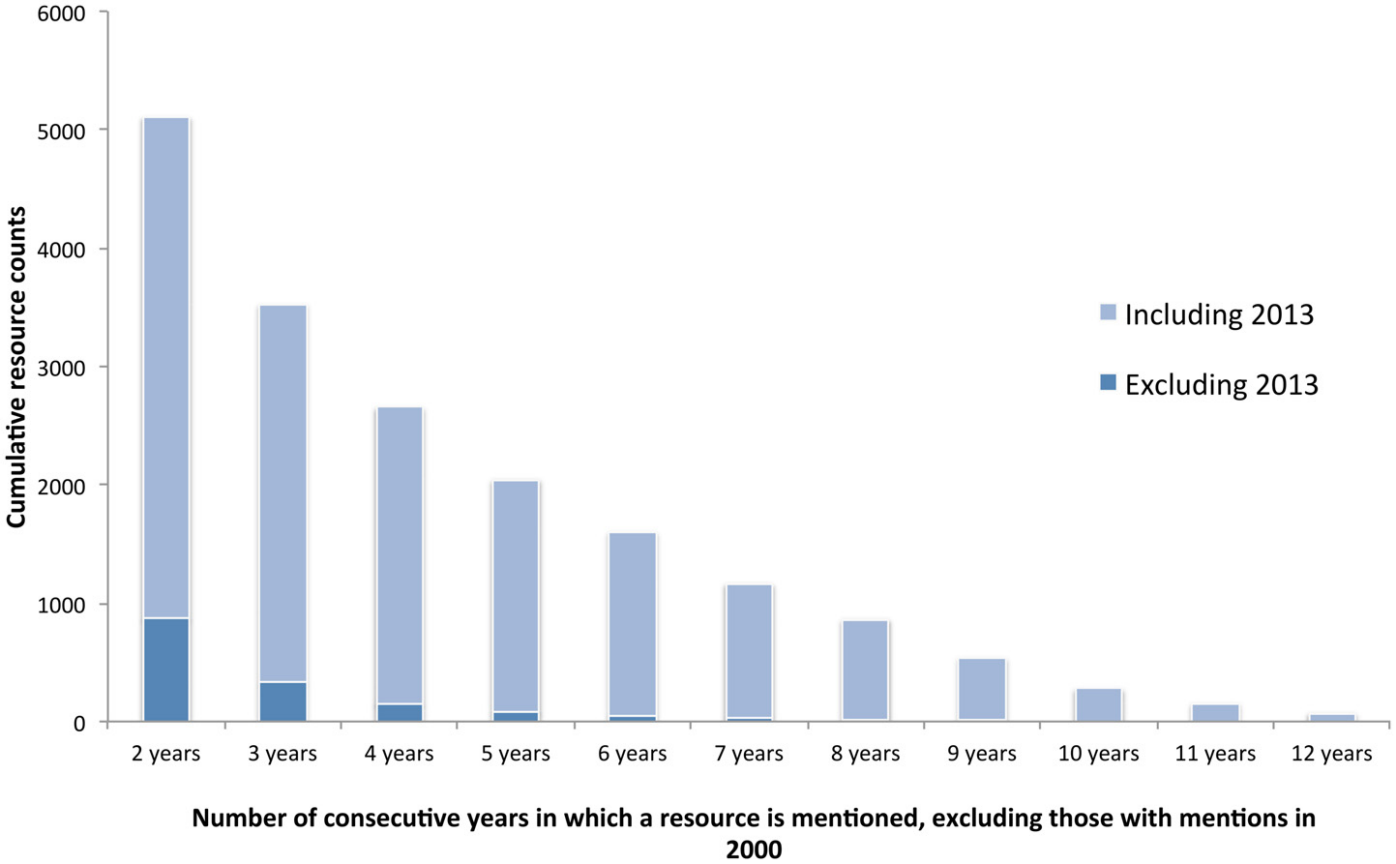
Cumulative number of resources that have persisted for a given number of years. The dark blue contains only resources that have not been mentioned in 2013, whereas the light blue contains resource mentioned in 2013. We excluded previously established resources by filtering out resources mentioned in 2000 (year zero).

Fig 8 provides a visualisation of these numbers. The dark blue provides the number of resources excluding those with mentions in 2013, and the light blue is the extra resources with mentions in 2013. This suggests that the majority of persistent resources first seen in the last decade, once established, remain in use today. In particular, there are over 1,500 resources that have been established in the last 5 years that are still used now. In addition, there is a relatively small number of persistent resources (56) that had continued usage runs for more than 5 years, which are no longer used. Given this, although these resources are not the well-established (more general) ones discussed earlier, they must still have some merit within some sub-domain if they successfully maintain persistence.

### Long-tail analysis

Previous research has reported that there is great variation in database and software use, with some gaining significant usage and citation, while others are not cited (and perhaps not used) at all [2, 24]. We wished to further investigate the varying levels of usage of different resources by analysing how often each resource extracted is mentioned at the document level within our *bioinformatics* corpus, and whether a few resources make up the majority of extracted mentions.

Our results enable us to see that a few well-established resources account for a large fraction of the total mentions, while many resources towards the end of the graph (the “long-tail”) are rarely (if at all) mentioned after their initial introduction. Specifically, 70% of the resource names we extracted from our *bioinformatics* corpus are only mentioned once each, and this 70% makes up only 11% of the total document level mentions we extracted. At the other end of the scale, a single resource (R) provides 4% and our top ten resource names make up for 18% of the total extracted mentions. Finally, the top 5% of resource names (133 names) account for a substantial 47% of the total extracted resources. This evidence not only confirms the pattern Galperin first saw [24], but shows that it also holds across the entire domain of bioinformatics. We further discovered that these patterns also hold true for all three of our other corpora, only the situation is generally worse—for example, a single resource is over 10% of the total mentions within *medicine* and over 7% for our full PMC set, and in each case 71–74% of resource names are only extracted from a single document.

### Comparison between different journals

Next we compared the proportion of resource mentions in different journals—this can help give an indication of where resources are reported (both first published, and then used). In particular, *PLoS ONE* has the most mentions, followed by *Nucleic Acids Research* and *BMC Bioinformatics* with 696,979, 269,875 and 203,882 total mentions each. If we instead sort by document level mentions, we again get *PLoS ONE* and Nucleic Acids Research (with 255,538 and 64,249 mentions), but *BMC Bioinformatics* is replaced by *BMC Genomics* (with 44,528 and 50,302 mentions respectively). The high numbers of mentions in *PLoS ONE* is indicative of the high volume of articles published in the journal (84,507 total articles within our corpus).

**Fig 9:**
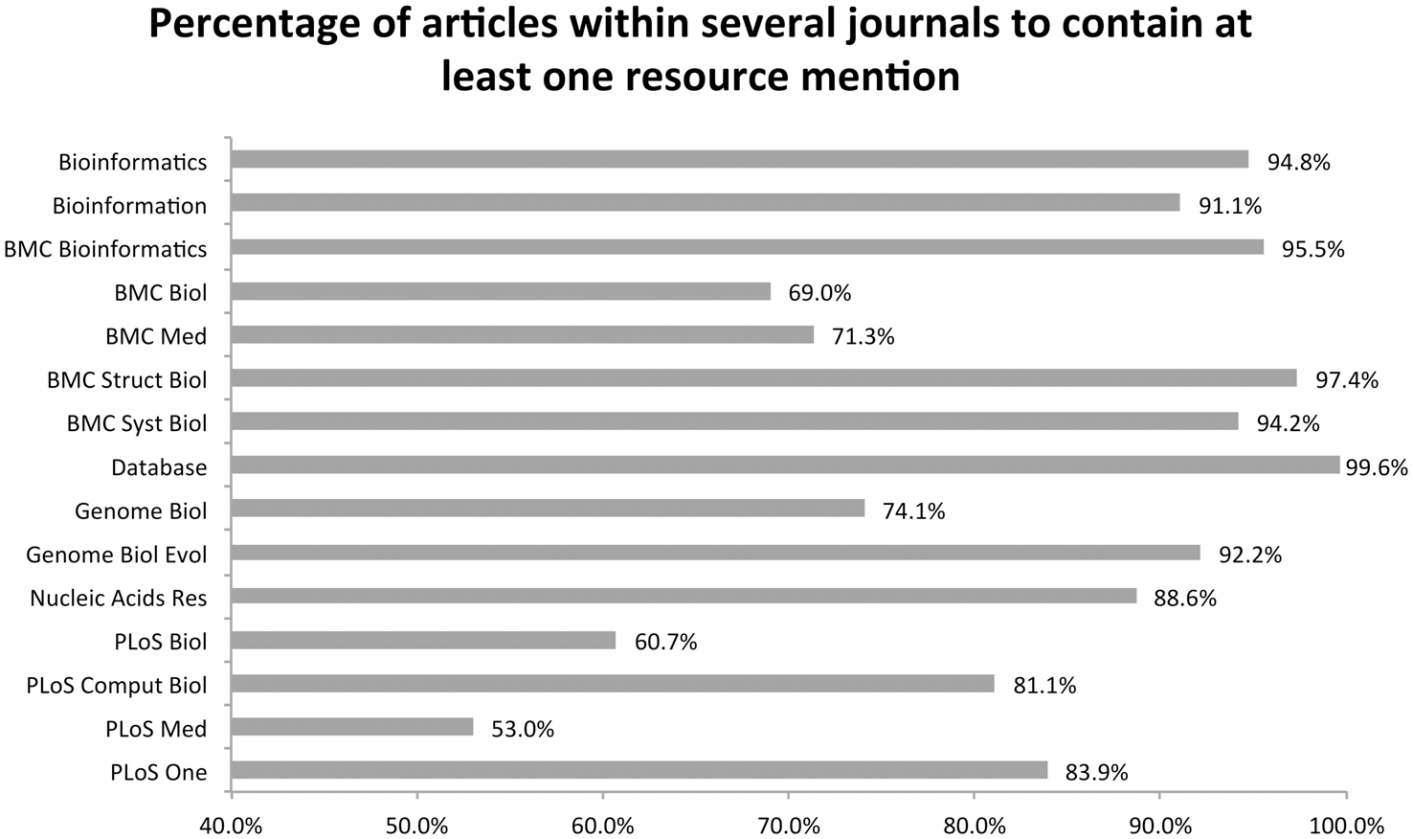
Comparison of journals based on the percentage of articles to contain a resource mention.

Finally, we ordered the journals in decreasing order of the proportion of mentions to documents, to see which journals were more resource rich, but ignored journals with fewer than 1000 articles to maintain a reasonable sample size. The resulting top ten journals, counts and proportions are provided in Tables 9 and 10. Additionally, we have calculated the percentage of documents within a journal to contain at least one database or software name mention (Fig 9).

**Table 9:** Journals with the highest proportion of total resource mentions.

Journal	Articles	Mentions	Proportion
BMC Bioinformatics	6,033	203,882	33.8
BMC Genomics	5,758	158,331	27.5
Bioinformatics	1,338	32,815	24.5
Nucleic Acids Res	11,121	269,875	24.3
BMC Syst Biol	1,067	24,461	22.9
BMC Plant Biol	1,381	25,787	18.7
Genome Biol	3,002	55,694	18.6
BMC Evol Biol	2,477	42,201	17.0
PLoS Comput Biol	3,095	47,614	15.4
Zookeys	1,419	19,729	13.9

Proportions are normalised by total article counts for each journal. This list is limited to only journals with at least 1000 articles within our corpus.

**Table 10:** Journals with the highest proportion of document level resource mentions.

Journal	Articles	Mentions	Proportion
BMC Genomics	5,758	50,302	8.7
BMC Bioinformatics	6,033	44,528	7.4
BMC Evol Biol	2,477	16,658	6.7
BMC Plant Biol	1,381	8,750	6.3
Bioinformatics	1,338	8,103	6.1
Nucleic Acids Res	11,121	64,249	5.8
BMC Syst Biol	1,067	5,837	5.5
Bioinformation	1,109	5,670	5.1
Genome Biol	3,002	14,973	5.0
PLoS Genet	4,029	18,366	4.6

Proportions are normalised by total article counts for each journal. This list is limited to only journals with at least 1000 articles within our corpus.

Several well-established journals have high percentages, and in particular *Database*, *Bioinformatics*, and *BMC Bioinformatics* have percentages over 94%. Interestingly, *BMC Structural Biology* and *BMC Systems Biology* also have numbers greater than 94%, which can be explained by their roots in bioinformatics. More “straight” biology based journals have lower percentages between 60 and 75% (e.g., *BMC Biology*, *Genome Biology* and *PLoS Biology*), and the same is true for medical based journals (e.g., *BMC Medicine* and *PLoS Medicine*). *PLoS ONE* has a surprisingly high percentage (84%) given it has a more general domain based focus.

### Journal and resource clustering

We investigate any separation between journals based on the resources mentioned within them, and between resources based on the journals in which they are mentioned, enabling us to characterise the resources by the journals in which they are mentioned, and the journals by the resources that they mention. We do this by performing a singular value decomposition (SVD; see Methods). This analysis could, for example, enable suggestions for resources to use based on the journal being targeted for paper submission, or provide suggestions for journals based on the resources a particular analysis has used.

**Fig 10:**
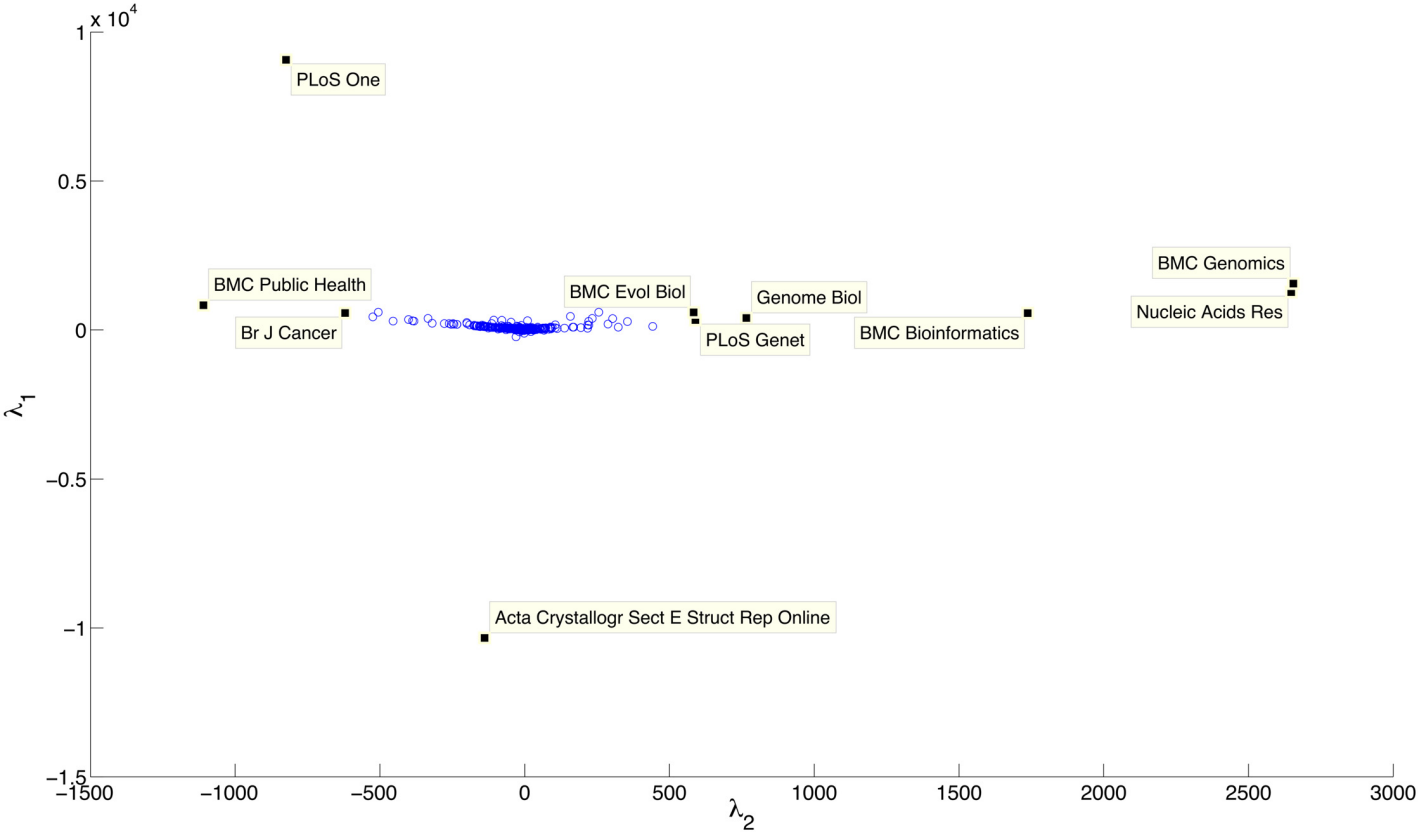
Plot of the two most important eigenvectors for journals, based on the resources contained within them. The x-axis appears to separate medical journals from bioinformatics based journals. The y-axis separates out two outliers—PLoS ONE (which is a extreme multi-disciplinary journal), and Acta Crystallography (which contained unusually frequent false positive mentions of R and SMART).

By plotting the two most important eigenvectors against each other (accounting for 19% of the total information variation; Fig 10), our analysis provides a separation that appears to segregate medical journals (left) from bioinformatics journals (right). This suggests that the x-axis vector separates different domains as each contains differing resource mentions. In addition, it separates PLoS ONE (up) and Acta Crystallography (down) from the others. This is likely to be because these are very different journals in terms of the resources they mention—PLoS ONE is a less domain specific journal covering a wide variety of subjects, whereas Acta Crystallography seems to contain an unusually high level of false positive mentions (on manual investigation) of R and SMART. As such, the y-vector may segregate journals by the range of resources contained within them; PLoS ONE has *many* resources with *many* mentions, whereas Acta Crystallography has *few* resources with *many* mentions (and few other resource mentions).

The large central cluster contains the majority of the journals used within this analysis. This is going to include journals that are hard to classify by the resources they contain. For example, journals with few mentions of resources, or other domain focussed journals—in particular, domains with lower (e.g., chemistry) or mixed (e.g., biology) resource usage.

**Fig 11:**
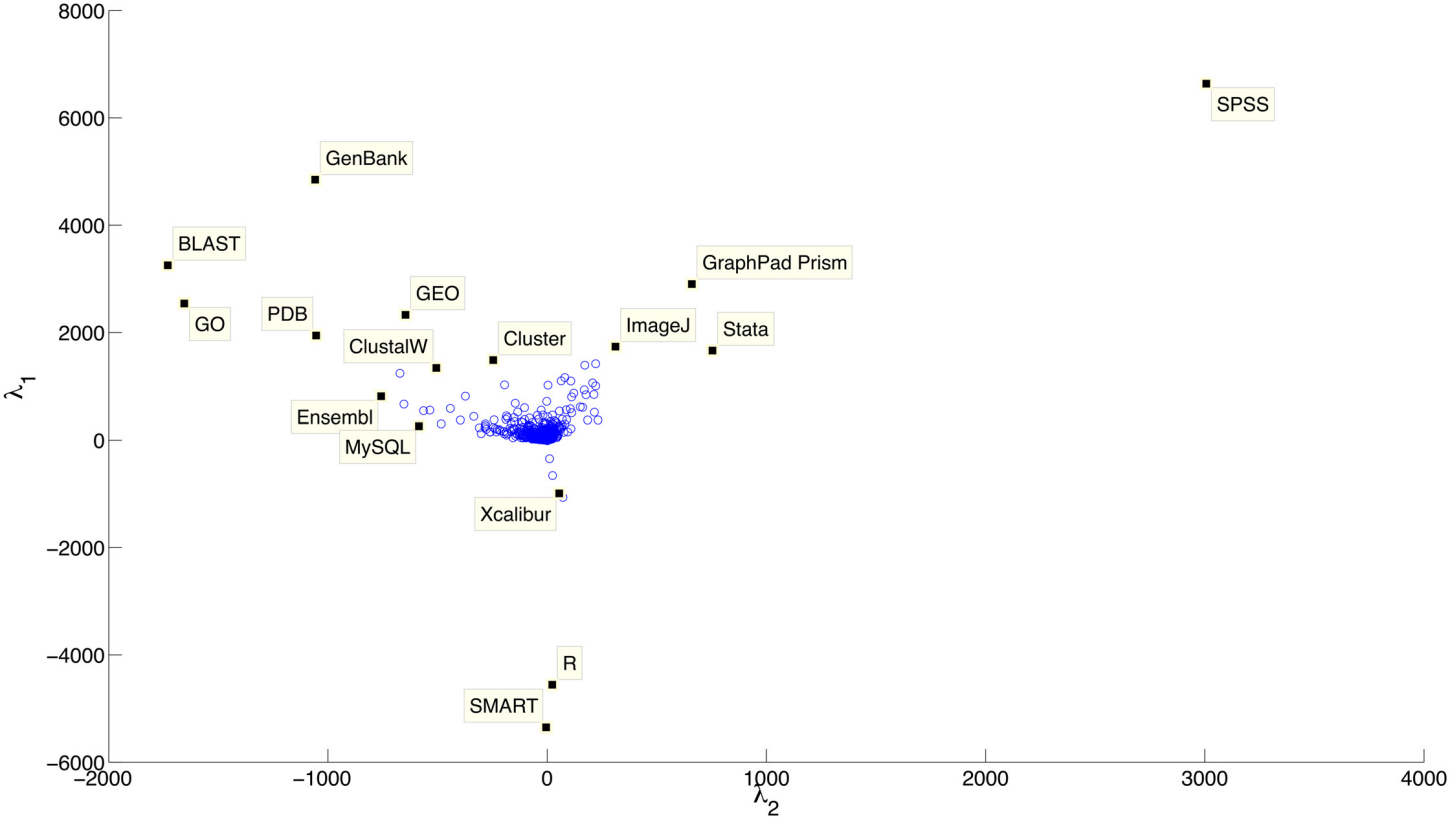
Plot of the two most important eigenvectors for resources, based on the journals they are mentioned within. The x-axis appears to separate bioinformatics resources from statistical software, whereas the y-axis appears to separate out SMART and R with some mass-spectroscopy tools (perhaps because these were pervasive false positives within Acta Crystallography).

Doing the same SVD analysis for resources by using the transpose of the previous matrix, we can cluster resources by the journals in which they are mentioned (Fig 11). There is a distinction between common/established bioinformatics resources (left) and statistics based software (right)—this probably relates to some journals having a greater focus on statistical analyses which are outside the more general bioinformatics domain (e.g., clinical studies). The other direction appears to be organised in an unusual way. The lower resources (R and SMART) are split from the others (SPSS, GenBank, BLAST), and are instead arranged close to some mass-spectroscopy (protein structure analysis) tools (e.g., Xcalibur). This could be highlighting a cluster of protein structure analysis tools, which contrast the genomic type resources such as GEO, GO, GenBank and BLAST along the same axis (up). This suggests that several journals focus on genomics whereas several others instead focus on proteomics.

The large central cluster contains resources that are either too general in terms of domain or are mentioned in too few journals to cluster. With further refinement, this could provide a way to recognise resources with similar functions across domains.

This analysis helps confirm our previous assessment, that *medicine* does indeed use different resources to *bioinformatics*, with *biology* frequently sitting in the centre of these two other disciplines. This could suggest that many bioinformatics resources stay contained within that domain, with some making an impact in biology, and very few finding their way into medical analyses. In addition, it suggests that proteomics has favoured resources infrequently seen within the rest of the literature, and that statistical programs such as Stata and GraphPad Prism have focused roles separating them from much more prolific tools such as BLAST and PDB.

### Resource usage within different paper sections

Unsurprisingly, the methods section generally has the highest number of resource mentions. There are relatively few differences between the sections in terms of resource names, although there are between corpora as previously discussed. That said, the GO seems to rank higher within the results/discussion section than in the methods—perhaps because it is more frequently used for annotation than for selection; and PubMed appears higher within the introduction—perhaps in reference to related literature and previous work. Also, as previously discussed, *bioinformatics* has consistently higher resource mention counts. Note that we are careful not to compare the relative counts of resources between sections as we have only normalised by the number of documents within each corpus, and not by the mean length of each section. The top ten document level resource mentions for each of the four main paper sections (introduction, methods, results/discussion and conclusion), within each of our four corpora, is provided in S1 Table.

### Resource usage within article captions

Our full PMC corpus has 77% of papers that contained at least one caption, and 22% of those with a caption contained a database or software name mention (only 17% of our total corpus). We extracted 459,534 resource mentions, with 199,890 total document level mentions. The most common resources we see within captions are actually very similar to those within full-text. Common, established resources such as BLAST, GenBank and ClustalW all make an appearance. That said, there is a slight preference for visualisation and image based resources too, as as may be expected. For example, ImageJ, the Protein Data Bank (PDB) and PyMol are all mentioned frequently. This preference is perhaps not as significant as expected, because we make no distinction about the type of caption we analyse—we equally analyse table, figure and supplementary captions.

### Limitations

Throughout this paper, we have assumed that resource usage is directly related to the number of resource mentions within text. Although a mention of a resource most commonly does imply its use, this is not always the case. For example, a resource could be mentioned as a comparison or an alternative to another, but not actually used within an analysis. To evaluate the extent of this occurrence within the literature, we have manually annotated our evaluation set of 25 full-text articles (see methods) to analyse resource usage verses “passing mention”. Specifically, if a resource is used for data generation, analysis, storage, etc., it is annotated as ‘used’. Equally, if a *quantitative comparison* is being made between resources, it is marked as ‘used’. However, if authors suggest that their resource can do something that another cannot (for example), this is not usage (e.g., information that could be gained from a resources associated publication or documentation, rather than by having to use the resource itself).

From these 25 articles, only 5.6% of the total mentions, and 5.2% of the document level mentions, were not of usage. The majority of these mentions (59%) were in the background or introduction section of a paper (provided as possible alternatives, for example). This suggests that around 95% of resource mentions in the literature are of usage, rather than reference.

Conversely, the total level of usage of a resource is likely to be much higher than is implied by its mentions alone. For example, a resource could have been tried but not used in the final analysis pipeline, used but not mentioned because it is “assumed knowledge”, indirectly implied (e.g., through a citation or through another resource), or otherwise deemed unnecessary [25, 26].

Despite these assumption and potential limitations, the patterns that we have found are still likely to hold given the complete usage of a given resource, and should not adversely affect our conclusions.

We have also made no distinction between the different types of resource found within the literature. These resources could reasonably be split into four distinct groups:

1. Databases—these form the primary data repositories within bioinformatics
2. Software—these are the primary sources of data analysis and manipulation
3. Packages—these are generally much smaller programs each with a specific purpose, often extending existing software or packages
4. Ontologies—these are the primary data annotation mechanisms.

These four resource types should perhaps be treated differently as they are likely to be reported in different ways within the literature. For example, databases might be reported by identifiers more often than by name, and packages might be reported instead of their primary software (or vice-versa) [25, 27]. Ontologies are often included within other software and other ontologies to enable annotation, and again might not be mentioned directly in text as a result. This links directly to our previous limitation as mentions do not equate directly to usage, though there is likely to be a sufficient correlation in our data on resource mentions to enable us to have appropriate answers to our questions.

## Discussion

We have demonstrated that a survey of the bioinformatics software and database landscape can be performed through the use of our named entity recogniser bioNerDS, and the data provided can be used to make observations about the state of the bioinformatics “resourceome”. We have automatically extracted bioinformatics database and software mentions from the full-text literature in PMC.

A common limitation of automated recognition software is that of false positive detection (i.e., low precision). This is a particular issue with resource recognition, due to the vast number of ambiguous resource names in use [22]. Well established resources such as MUSCLE, R and BLAST could produce false positive hits, and lesser known resources such as the R packages analysis, genomes and cell are likely to generate more false positive than true positive hits within general biomedical text (regardless of case sensitivity). Acronyms naturally causes further ambiguity (e.g., GO, ECG)—especially when they are also used in database identifiers (e.g., GO:0005554)—but can potentially be mitigated by more complex acronym resolution techniques [28]. bioNerDS attempts to minimise false positive results by recognising negative keywords surrounding potential resource names, and by requiring a minimum positive threshold before a term is accepted. Extra work has been undertaken to further minimise false positive recognition by incorporating a machine learning based post-processing filter into the bioNerDS pipeline. This filter separately scores only accepted terms, upon which an additional filter may be customised and used. The stricter the threshold, the fewer false positives generated by the resulting bioNerDS system (though there is always the inevitable trade-off in recall to consider).

Through a comparison in resource usage between the *medicine*, *biology* and *bioinformatics* domains, we conclude that bioinformatics’ reputation as a resource dependant domain (or *resourceome*) is well founded. The biology corpus contained the highest proportion of total resource mentions within its articles. We also find that, as one would expect, resource focused journals have generally higher resource mention counts.

Our results highlight the diverse and dynamic nature of bioinformatics research. There are clear differences in resource usage within different domains. For example, bioinformatics appears to prefer R, whereas researchers working within medicine prefer SPSS for statistical analysis, while biologists use both with no clear community preference. In addition to domain trends, several resources are prevalent within all fields, for example, well-established “house-hold” names such as the Gene Ontology and GenBank. In particular, GO, GEO and R have seen significant growth in relative usage over the last ten years within bioinformatics, becoming core resources in patterns of database and software use [16].

We also evaluated the differences between document sections with regards to the levels of resource mentions, and find, not surprisingly, that the methods section contains the highest number of resource mentions within all four of our corpora. We provide evidence that resource mentions in document captions, based on the database and software names mentioned within them, are frequently used to describe the visualisation software used.

Though we note that many of the results we provide within the paper are perhaps to be expected, for example, popular bioinformatics resources, this is the first time where such knowledge has been successfully quantified computationally on such a large scale. In particular, our results corroborate a previous survey of resource usage within the bioinformatics domain [9]. This is encouraging as it suggests resource usage (and thus potential method) consistency within the bioinformatics domain (across journals), here using a substantially larger underlying dataset. In addition this work evaluates and contrasts bioinformatics to the biology and medicine domains, which provides further insight that was not previously available.

Our results show that just the top 5% of resource names account for 47% of total usage. We also find that resources can become established, even if they are not frequently used, suggesting the importance of resources to more specialised or niche areas of research activity. This notion of persistent utility does assume, however, that resource persistence implies direct usefulness. For example, perhaps a tool is still in use because of its ease of installation, usability, or inter-operation, even if the results are sub-optimal [29]—though it can be argued that it still has some utility in this case. Alternatively, these could be closed or prohibitively expensive resources resulting in them not being reused extensively. Possibly it is these low-mention but persistent resources that need identifying and specific support, despite not being core to the bioinformatics resource using community as a whole.

That over 70% of resource names are only mentioned once implies much wasted effort on behalf of those developing bioinformatics databases and software. A developer or institution’s ability to maintain and support a resource can be, however, influenced by a number of factors, not least of which is funding, but also includes the software and data management practises of the community [30]. This has potential implications in resource sustainability, and though we cannot reasonably expect all resources ever created to become widely used and established, increased awareness and use of good software engineering and data curation practices should increase sustainability and perhaps be part of funding models; see, for instance Software Carpentry [30] and Data Carpentry (http://datacarpentry.org/). Some of these single mention resources may earn their place through increased usage as a result of better engineering that makes them usable, maintainable, robust and so on [30, 31]. Even if a resource “dies”, the insights gained from any resource development will generally persist, even if the resource itself does not—the additional knowledge gained through increased competition is likely to be a good thing; the long-tail is likely to be where more innovation in software takes place [29].

Encouraging the community to accept a newer (and potentially “better”) resource as an alternative to another could be considered as much a sociological issue as it is dependent on the research, development, sustainability and marketing of the alternative resource. Our survey shows that the bioinformatics resource profile is dynamic, with resources being replaced by others and there is much innovation around a slowly changing core. While rapid change is necessary for progress, there is a danger that useful innovations may be lost, resulting in a necessity for “reinventing the wheel”. A move towards platforms such as R and Galaxy that allow innovation within them, which have become much more prominent, may mean that co-ordination, dissemination and promotion become easier. In a wider context, the developments of platforms that become core to an activity via openness and interoperability, and that enable third party innovation is seen as the route towards those resources reaching a tipping point that enables them to become core to that activity [32].

In conclusion, the survey we have performed is a necessary step towards being able to judge how much a computer-based resource is being used; when a resource is rising in use, falling out of use, either globally or within its niche domain, or being superseded by another. For example, our results hint at a steady uptake in the use of MUSCLE, while usage in ClustalW has declined. Such knowledge could be useful for determining best-practice, depending on the actual metrics used [8], and could enable targeted and sustainable support of important resources by providing an overview of resource usage within the published literature (especially where citations alone may not provide the full picture). Our name entity recogniser, bioNerDS, can be used to survey literature for mentions of bioinformatics databases and software and can provide insights into the population and its dynamics in the bioinformatics resource ecosystem.

The full dataset generated and used for this study is free to access and reuse under the CC0 license here: http://dx.doi.org/10.6084/m9.figshare.1281371.

## Supporting Information

S1 Table Top ten document level resource mentions within different document sections within our corpora. The numbers provide the relative *percentage* of each corpus to contain a mention of that resource. For example, 1.72% of Full PMC introduction sections contain a mention of R. Note that *ECG* features twice within the *medicine* tables, as it was in there once in its expanded and once in its acronym form (which has been manually corrected for readability in the final table)—our automated variant aggregation failed to combine these into a single final entry on this particular occasion.
(XLSX)
